# Serum Visfatin Levels, Adiposity and Glucose Metabolism in Obese Adolescents

**DOI:** 10.4274/Jcrpe.547

**Published:** 2012-06-09

**Authors:** Derya Taşkesen, Birgül Kirel, Tercan Us

**Affiliations:** 1 Eskişehir Osmangazi University Hospital, Department of Pediatrics, Eskişehir, Turkey; 2 Eskişehir Osmangazi University Hospital, Pediatric Endocrinology Unit, Eskişehir, Turkey; 3 Eskişehir Osmangazi University Hospital, Department of Microbiology, Eskişehir, Turkey; +90 222 229 00 64+90 506 713 80 18birkirel@yahoo.com

**Keywords:** Visfatin, Obese, insulin resistance, glucose tolerance test

## Abstract

**Objective:** Visfatin, an adipokine, has insulin-mimetic effects. The main determinants of both the production and the physiologic role of visfatin are still unclear. The aim of this study is to determine the relation of serum visfatin to adiposity and glucose metabolism.

**Methods:** 40 pubertal adolescents (20 females, 20 males; age range: 9-17 years) with exogenous obesity and 20 age- and sex-matched healthy adolescents (10 females, 10 males) were enrolled in the study. Oral glucose tolerance test (OGTT) was performed in the obese group. Serum glucose, insulin and visfatin levels were analyzed in the fasting state in the controls and at 0, 60 and 120 minutes during the OGTT in the obese group.

**Results:** The obese group had higher serum visfatin levels than the control group [11.6 (3.3-26) ng/mL vs. 7.5 (3.3-10.5) ng/mL, p<0.001]. Visfatin levels were correlated positively with body mass index, waist/hip ratio, insulin, and homeostasis model assessment for insulin resistance and negatively with glucose/insulin ratio in the combined group (obese subjects plus controls). Visfatin levels were essentially similar in obese subjects with and without insulin resistance (p>0.05). Serum visfatin levels did not change at 60 and 120 minutes of the OGTT compared to the baseline levels (p>0.05).

**Conclusions:** Serum visfatin levels are elevated in obese adolescents and do not change with acute changes in glucose metabolism. Visfatin levels are related with adiposity and glucose metabolism parameters. However, the role and contribution of adiposity and glucose metabolism to the circulating visfatin levels in obese patients remain to be explored.

**Conflict of interest:**None declared.

## INTRODUCTION

Visfatin was previously known as the pre-β cell colony-enhancing factor (1). In 2005, Fukuhara et al (2) showed that this substance is also an adipocytokine produced predominantly by human visceral adipose tissue and that its plasma levels are related to adiposity. Visfatin exerts insulin-mimetic actions through insulin receptors. Acute administration of visfatin to mice lowered glucose levels (2). Mice with chronically elevated plasma visfatin levels had decreased levels of both glucose and insulin, and plasma glucose levels were higher in mice heterozygous for visfatin deficiency than in normal mice (2). In addition, visfatin had effects on lipid homeostasis similar to that of insulin and it was also involved in adipocyte proliferation and differentiation and triglyceride (TG) metabolism (2). Based on this knowledge, circulating visfatin levels and its determinants have been investigated in several clinical studies (3,4,5,6,7,8,9,10). The aim of this study was to determine the relation between serum visfatin levels and adiposity in obese and non-obese adolescents. The relationships between visfatin levels and parameters of glucose metabolisms were also investigated.

## METHODS

Of patients attending our endocrinology outpatient clinic, 40 obese adolescents (20 females, 20 males) aged 9-17 years were included in this study. Known pathological causes of obesity were ruled out and all patients were diagnosed as having exogenous obesity. Twenty age- and sex-matched healthy adolescents (10 females, 10 males) served as a control group. All subjects in the study showed signs of puberty (Tanner stages 2-5) (11). No patient was receiving any medication for any reason.All subjects were examined by the same physician. Blood pressure was also recorded. Puberty was staged according to breast development in girls and testicular volume in boys. Body weight (BW), waist and hip circumferences were measured by standard methods and devices. 

Waist/hip ratio (WHR) was calculated for each subject.

Body mass index (BMI) was calculated by the equation: body weight (kg)/height (m2). Obesity was defined as a BMI value higher than the 95th percentile value for age and gender according to reference values for Turkish children (12). BMI standard deviation score (SDS) was calculated using the SDS Individual Calculator for British 1990 Growth Reference Data by Mark Delderfield (13).An OGTT was performed in all subjects in the obese group. For this test, following a 3-day carbohydrate-rich diet and an overnight fast, the subjects ingested an oral dose of 1.75 g/kg (maximum 75 g) glucose. Venous blood samples were collected at 0, 60 and 120 minutes for determination of serum glucose, C-peptide, insulin and visfatin levels. The serum samples at 0 minute of the OGTT were also assessed for serum lipid and lipoprotein levels. The OGTT results were evaluated according to the World Health Organization (WHO) criteria (14).In the control group, after an overnight fast, venous blood samples were taken for determination of serum visfatin as well as glucose and lipid metabolism parameters.

Serum samples for visfatin were stored at -80°C until the analysis was performed. Serum visfatin levels were determined using a commercial ELISA kit (Phoenix Pharmaceuticals, Inc. Belmont, CA) in our microbiology laboratory. Serum levels of glucose, insulin, C-peptide, triglycerides (TG), total cholesterol (TC), high-density lipoprotein cholesterol (HDL-C) and low-density lipoprotein cholesterol (LDL-C) were determined in our biochemistry laboratory, using standard methods.

Homeostasis model assessment for insulin ratio (HOMA-IR) was calculated by the following equation: fasting glucose (mg/dL) x fasting insulin (μcU/mL)/405) (15). The HOMA-IR values were compared with the cut-off point value defined for Turkish adolescents, and a HOMA-IR value higher than 3.16 was accepted as IR (16). Glucose/insulin ratio was calculated from the fasting levels of these parameters. In accordance with the values given by the International Diabetes Federation (IDF) for adolescents, a TG level exceeding 150 mg/dL was accepted as high and a HDL-C value lower than 40 mg/dL was accepted as a low HDL-C level (17).

Statistical analysis was performed using PASW 18 and SigmaStat 3.5. According to the results of normality tests (Shapiro-Wilk), visfatin levels were not normally distributed. Non-parametric statistical tests were used for the analyses related to visfatin. The data were given as median and min-max values. Friedman repeated-measures analysis of variance on ranks was used to test the significance of changes in serum glucose, C-peptide and visfatin levels during OGTT. Non-parametric Tukey method was used for the post hoc multiple comparisons. Independent-samples t-test and Mann-Whitney U test were performed for the other comparisons. Spearman’s rank correlation test was used for the simple correlations between visfatin and other variables. Probability values of p<0.05 were considered as significant.The study protocol was approved by the Eskisehir Osmangazi University Ethics Committee. The children and their parents were informed about the objectives and methods of the study. Informed consent was obtained from all parents and also from the adolescents.

## RESULTS

The obese group had higher BW, BMI, WHR and HOMA-IR values, higher fasting C-peptide and insulin levels, and lower glucose/insulin ratios than the control group (Tables 1 and 2). TG and LDL-C levels were also higher in the obese group than in the controls (p<0.001 for both). Eleven obese subjects had high TG. Low HDL-C was found in 14 obese and 3 control subjects. IR was present in 19 obese subjects. Diabetes mellitus was diagnosed in 2 patients who had both impaired fasting glucose values and glucose levels higher than 200 mg/dL at 120 min of OGTT. Impaired glucose tolerance was determined in 2 obese subjects. Hypertension was not detected in any of the subjects.Fasting serum visfatin levels (at 0 min) in the obese group were significantly higher than in the control group (p<0.001) (Table 2). No gender difference was found in serum visfatin levels in either group (p>0.05).

During the OGTT, glucose, C-peptide and insulin levels increased and were found to be significantly higher at 60 min compared to baseline levels (p<0.001) (Table 2). These levels decreased at 120 min. However, there were no statistically significant differences between the values at 60 min and 120 min (p>0.05). The levels of all these parameters were significantly higher at 120 min than at 0 min (p<0.05).Serum visfatin levels did not change significantly from the initial values (at 0 min) at 60 and 120 min of the OGTT (p>0.05). There were also no significant differences between the levels at 60 min and 120 min of the OGTT (p>0.05). Visfatin levels at 0 min did not show any differences between the obese subjects with and without IR (p>0.05).

When simple correlation analysis was performed, fasting visfatin levels were not correlated with either glucose, insulin and C-peptide levels, visfatin levels at 60 and 120 min of the OGTT, or any other variable in both the obese and control groups (p>0.05). However, in the combined group (obese subjects plus controls), visfatin levels were strongly correlated with BW, BMI, WHR (r=0.4, r=0.5, r=0.5, respectively, p=0.001 for all), TG and LDL-C levels (r=0.3, p<0.05 for both). Visfatin levels were correlated positively with insulin, C-peptide, and HOMA-IR (r=0.45, p=0.001; r=0.4, p<0.001; and r=0.4, p=0.01, respectively), and negatively with glucose/insulin ratio (r=0.4, p=0.002) in the combined group. 

## DISCUSSION

Since visfatin is produced mainly by adipocytes (2), it is expected that the concentration of the circulating visfatin increases with an increase in the amount of body fat. In the study by Fukuhara et al (2), KKAy mice, a model for obese type 2 diabetes, were fed with a high-fat diet. As the mice became obese, plasma visfatin levels gradually increased with time and visfatin mRNA levels also increased in the visceral adipose tissue and in the liver of these mice. Furthermore, it has been reported that weight loss with a hypocaloric diet for three months and bariatric surgery resulted in a decrease in circulating serum concentrations of visfatin in obese subjects (18,19). Concordantly, we found higher serum visfatin levels in obese adolescents as compared to normal-weight subjects. Similarly, in most clinical studies, circulating visfatin levels were found to be elevated in obese children (3,4,5,6,7) and adults (20,21,22) as well as in subjects with metabolic syndrome (5,23). However, in most of these studies, elevated visfatin levels were not related with adiposity parameters such as BMI, WHR and percentage of body fat (6,7,20,21). In our study, serum visfatin levels were not correlated with BW, BMI and WHR in either obese or control groups. However, there were correlations between serum visfatin and these parameters in the combined study group (obese and control subjects). These findings suggest that adiposity is related with circulating visfatin, but only if there are remarkable differences for adiposity among the study subjects.

Studies on relation of circulating visfatin to adiposity have yielded variable results. Davutoglu et al (3) found elevated plasma visfatin levels in obese children and have reported that these levels were positively correlated with BW, BMI, as well as waist circumference and WHR. In the study by Berndt et al (24), plasma visfatin concentrations and visfatin mRNA in visceral adipose tissue were positively correlated with BMI and body fat content in subjects with a wide range of obesity. Pagano et al (25) reported reduced plasma visfatin levels and visfatin mRNA expression in subcutaneous adipose tissue in obese adults. These authors found a negative correlation between plasma visfatin level and BMI. In this study, since BMI could explain only 14% of the variability of plasma visfatin in obese subjects, the authors suggested that there must be some different sources of visfatin other than the adipose tissue. We also think that as adiposity is a factor related to circulating visfatin, its role and contribution need to be clarified.

In our study, fasting serum visfatin level was not correlated with the glucose metabolism parameters in both the obese and control groups. On the other hand, serum visfatin level was correlated with serum levels of glucose, insulin and C-peptide, as well as with HOMA-IR in the combined group. Also, there was a negative correlation between serum visfatin and glucose/insulin ratio in this combined group. As the results of our study also demonstrated, most clinical studies failed to show any association of circulating visfatin with fasting glucose, insulin levels, HOMA-IR (5,8,9,26,27,28), and insulin sensitivity (23,29) in either healthy subjects, obese subjects or diabetics. However, Davutoglu et al (3) found positive correlations of circulating visfatin to HOMA-IR and insulin levels. There are also conflicting results on the relationships between circulating visfatin and IR. In hypertensive adults, visfatin levels were reported to be higher in patients with IR than in those without IR. Independent of this finding, a relationship between serum visfatin and HOMA-IR was also reported (30). Araki et al (4) did not find any difference in circulating visfatin levels between obese adolescents with and without IR. Our findings are consistent with this report. According to other studies, treatment with hypoglycemic agents had no effect on circulating levels of visfatin in diabetic patients (31). These levels were also not affected by an increase in IR caused by dexamethasone use in obese subjects (28) and by lipid infusion-induced IR (29). Both Hofsø et al (10) and Jian et al (32) reported that circulating visfatin levels were not different among diabetics and subjects with impaired and normal glucose tolerance. On the other hand, elevated circulating levels of visfatin were reported in many studies on type 2 (9,26,27,29,31,33,34) and type 1 diabetics (29,35). In one study, which reported higher circulating visfatin levels in type 2 diabetics and in long-standing type 1 diabetics, visfatin levels were found to increase with beta-cell dysfunction and to correlate negatively with glycemic control in type 2 diabetics (29). In contrast, Toruner et al (36) reported low visfatin levels which correlated negatively with both glycemic control and disease duration in type 1 diabetics. Regular chronic exercise (35) and intensive glycemic control (34) lowered the high plasma visfatin levels in both type 1 and type 2 diabetics. These reports suggest that visfatin may play a role in glucose metabolism.

In our study, the concentration of serum visfatin did not change during OGTT and was not affected by the changes in serum glucose and insulin levels. Similarly, Marcinkowska et al (28) reported no change in serum visfatin levels at 60 and 120 minutes during OGTT in obese adults. In the same study by these authors, administration of dexamethasone to another group for two days resulted in an increase in fasting insulin and HOMA-IR without a significant change in serum visfatin concentrations. According to these results, circulating visfatin is not affected by the short-term regulation of glucose abnormalities such as hyperglycemia and hyperinsulinemia and IR. In addition, Haider et al (37) showed that in healthy subjects, circulating visfatin levels increased with intravenous infusion of glucose in a dose- and time-dependent manner and this glucose-induced elevation of serum visfatin was prevented by co-infusion of insulin and somatostatin. In this study, human adipocytes were also cultured to study visfatin release and mRNA expression in vitro. Concentrations of visfatin in the supernatant media in this study model were increased by prolonged exposure to high concentrations of glucose.

During the OGTT, after an oral glucose load, blood glucose and insulin levels reach a peak level and then start to decrease to below the postprandial level of 140 mg/dL at 120 minutes. On the other hand, in the study reported by Haider et al (37), circulating visfatin levels increased during a glucose clamp maintained for 270 minutes with stepwise increased glucose concentrations of 5.0, 8.3 and 11.1 mmol/L for a period of 90 minutes each. So, it may be speculated that since glucose and insulin levels were not maintained at the same high level during the OGTT as in the study by Haider et al (37), the stimulus for the change in circulating visfatin level could not be maintained in this short duration of glucose elevation in the course of the OGTT. Thus, as suggested by Haider et al (37), circulating visfatin may be regulated by changes in glucose metabolism, but this relationship is dependent on the duration and magnitude of glucose elevation.Similar to our results, Fukuhara et al (2) did not find a change in plasma visfatin levels during fasting and fed states in mice, while plasma insulin levels increased in the fed state and decreased in the fasting state. In morbid obese subjects, Hofsø et al (10) described increased serum visfatin levels transiently at 30 minutes of the OGTT without any correlation with changes in glucose and insulin levels and independent of obesity and glucose tolerance status. Serum visfatin levels returned to baseline values at 120 minutes. These authors have suggested that glucose may be involved in the acute regulation of the visfatin.

While serum visfatin levels do not change with the acute changes in glucose metabolism, the positive correlations of fasting serum visfatin with glucose, insulin and HOMA-IR and the negative correlation between fasting serum visfatin and glucose/insulin ratio in our combined group of subjects suggest that serum visfatin level may be related with hyperglycemia and hyperinsulinism. The high levels of visfatin reported in diabetes also support the existence of such a relationship (9,26,27,29,31,33,34,35). Some authors have suggested that the elevation of circulating visfatin in diabetes is a compensatory attempt to lower chronic hyperglycemia (29,34,36). In the study reported by Fukuhara et al (2), acute administration of recombinant visfatin to mice lowered plasma glucose levels without any change in insulin levels. These authors also reported that chronic elevation of visfatin levels in mice caused a decrease in both plasma glucose and insulin levels (2). All these results, including the findings reported by Haider et al (37) , despite some conflicting reports, suggest that visfatin may have an as yet unexplained role in glucose metabolism.

In conclusion, our findings indicate that serum visfatin levels are elevated in obese adolescents and do not change with the acute changes of glucose metabolism. Visfatin levels in obese subjects do not appear to be related with adiposity and glucose metabolism parameters. On the other hand, a relationship between serum visfatin and these parameters was established in the combined group which included both obese and normal-weight subjects, a group with a wide range of adiposity. We believe that the source and determinants of serum visfatin in obese patients is an area which needs to be further explored. 

## Figures and Tables

**Table 1:**
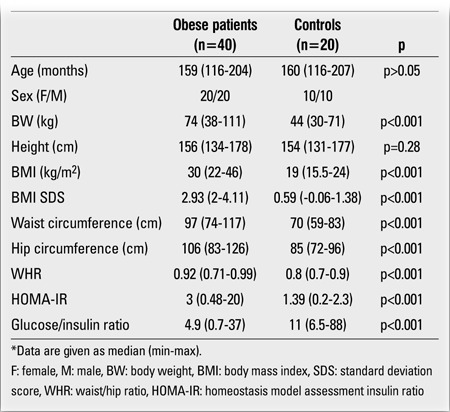
The anthropometric and baseline laboratory values of the studygroups*

**Table 2:**
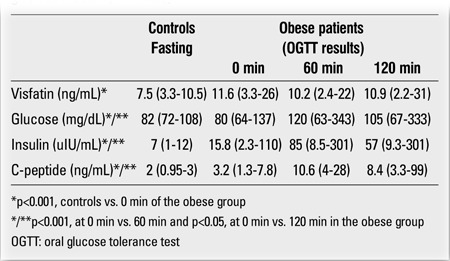
Biochemistry results in controls and obese patients [Data aregiven as median (min-max)]
